# Comparison of propofol fentanyl versus propofol ketamine for painless gastrointestinal endoscopy: a systematic review

**DOI:** 10.3389/fmed.2026.1946236

**Published:** 2026-09-21

**Authors:** Sheng Xiao, Ji Zhang, Shiyan Xiao, Qifu He

**Affiliations:** 1 Department of Anesthesiology, Wuhou District People’s Hospital of Chengdu, Chengdu, China; 2 Department of Oral Pathology Department, West China Hospital of Stomatology, Sichuan University, Chengdu, China; 3 Department of Science and Education Section, Wuhou District People’s Hospital of Chengdu, Chengdu, China

**Keywords:** colonoscopy, gastrointenstinal, ketamine, propofol, systematic review

## Abstract

**Background:**

Propofol-based sedation is widely used for gastrointestinal endoscopic procedures because of its rapid onset and recovery profile. However, adjunct agents are required to improve procedural tolerance and reduce propofol-related cardiorespiratory adverse effects. Ketamine and fentanyl are among the most commonly combined agents, but their comparative performance in gastrointestinal endoscopy remains unclear. This review compared propofol-ketamine with propofol-fentanyl for sedation during gastrointestinal endoscopy.

**Methods:**

Systematic search of major electronic databases was conducted from inception to March 2026. Study selection, data extraction, and risk-of-bias assessment were performed independently by reviewers using standard methodologies. Due to substantial heterogeneity in study population, procedure, sedation protocol, and outcome definition, findings were synthesized narratively. Outcomes of interest included additional propofol requirement, oxygen desaturation, total propofol dose, systolic blood pressure, mean arterial pressure, and time to recovery.

**Results:**

21 studies were included. Across included studies, propofol-ketamine required fewer supplemental propofol doses and lower overall propofol consumption than propofol-fentanyl. Oxygen desaturation tended to occur less frequently with propofol-ketamine in most studies, although definition varied. Hemodynamic outcomes favoured propofol-ketamine, with better control of systolic blood pressure and mean arterial pressure. Recovery findings were less consistent as several studies suggested longer recovery with ketamine-containing regimens, whereas other studies found no difference.

**Conclusion:**

Propofol-ketamine might provide better respiratory and hemodynamic stability and propofol-sparing effect compared with propofol-fentanyl during gastrointestinal endoscopy, although recovery may be slower in fewer settings.

## Introduction

Gastrointestinal endoscopy is one of the most frequently performed diagnostic and therapeutic procedures in modern clinical practice (1). Adequate sedation has become central for its safe and acceptable delivery. At althe same time, endoscopic sedation must balance competing goals like adequate hypnosis and analgesia, preserving cardiorespiratory stability, rapid recovery, and efficient turnover in busy endoscopy units (2). Recent guidance and reviews continue to emphasize that ideal regimen for endoscopic sedation remains context-dependent (3, 4). It should be individualized according to patient risk, procedural complexity, and available expertise (1–4).

Propofol has emerged as dominant sedative for gastrointestinal endoscopy due to its rapid onset, predictable titratability, and short recovery profile. These characteristics makes it better for ambulatory endoscopy, as quick induction and discharge are highly valued in such setting. However, propofol is not analgesic, and when used alone it may require higher doses to suppress any procedural discomfort and movement, especially during more stimulating procedures. Dose escalation can, in turn, increase risk of respiratory depression, oxygen desaturation, hypotension, and deeper than intended sedation. These limitations have caused continued interest in combining propofol with any adjunct agents that will be able to reduce total propofol exposure while maintaining procedural conditions and patient safety (1–4).

Fentanyl has been used as one such adjunct for longer period of time due to its potent analgesic properties and also familiarity in endoscopy practice. Nevertheless, opioid-based regimens carries disadvantages such as dose dependent respiratory depression, hypoventilation, oxygen desaturation, and delayed recovery in susceptible patients. These concerns are more relevant in endoscopy, where airway access might be limited during procedure and many patients are older or might have cardiopulmonary comorbidity. Hence, endoscopy guidelines and sedation reviews continue to stress that there should be careful patient selection, monitoring, and relevant drug choice whenever opioids are incorporated into propofol based sedation (1, 3, 4). Ketamine offers different pharmacologic profile that makes it appealing alternative in combination with propofol. Unlike opioids, ketamine provides analgesia together with dissociative and sympathomimetic effects, and it can preserve airway reflex and preserve blood pressure more effectively than pure hypnotic opioid combinations. Hence, this combination has gathered more attention across procedural sedation setting, including gastrointestinal endoscopy (3, 5–7).

However, ketamine is not without limitations. There were concerns regarding psychomimetic effects, increased secretions, nausea and vomiting and sometimes prolonged recovery depending on dose and admixture ratio. Recent research in colonoscopy and gastrointestinal endoscopy have suggested that ketamine containing regimens may reduce propofol consumption and provide more stable hemodynamic, while opioid containing regimens may offer simple recovery characteristics. However, findings across studies have not been entirely uniform (7–9).

This research question has become more important as endoscopy services expands and sedation practice has been shifting towards precision, safety, and efficiency. Recent reviews have highlighted that propofol remains benchmark sedative for endoscopy, yet choice of adjunct analgesic continues to vary considerably across institutions and practitioners (2–4). Broader literature also suggests that ketamine containing strategies might offer favourable respiratory and satisfaction profiles in certain settings, while safe implementation of any regimen depends on appropriate monitoring standards, competence of clinician, and readiness to manage any adverse events (5, 8, 10). However, more focused evaluation of propofol ketamine combination against propofol fentanyl combination in gastrointestinal endoscopy is necessary to address practical decisions faced daily in endoscopy units. Hence, this review was done to compare propofol fentanyl against propofol ketamine for painless gastrointestinal endoscopy.

## Methods

### Study design and reporting standard

This review was conducted and reported in accordance with Preferred Reporting Items for Systematic Reviews and Meta-Analyses (PRISMA) 2020 statement (11).

### Eligibility criteria

Studies were eligible if they met all the following criteria (framed based on PICOS framework).

#### Population

Patients of any age group undergoing gastrointestinal endoscopic procedures under procedural sedation including but not limited to upper gastrointestinal endoscopy, colonoscopy, and other diagnostic/therapeutic endoscopic procedures.

#### Intervention

Sedation regimens using propofol and ketamine combination.

#### Comparator

Sedation regimens using propofol and fentanyl combination.

#### Outcomes

Studies reporting at least one clinically relevant sedation-related, respiratory, hemodynamic, drug-consumption, or recovery outcome relevant to this review. The outcomes of interest included: requirement for additional propofol doses in the first minute, oxygen desaturation, total propofol dose, systolic blood pressure, time to recovery, and mean arterial pressure. Other closely related intra-procedural safety and recovery variables reported by eligible studies were also extracted where relevant to contextualize the findings.

#### Study design

Randomized controlled trials and comparative prospective or retrospective studies directly comparing propofol–ketamine with propofol–fentanyl were eligible. Non-comparative studies, case series, case reports, conference abstracts without sufficient data, reviews, editorials, letters, animal studies, and simulation studies were excluded.

### Information sources

A comprehensive literature search was performed in the following electronic databases: PubMed/MEDLINE, Scopus, Embase, Cochrane CENTRAL, and Web of Science from database inception to March 2026. In addition, the reference lists of all included studies and relevant review articles were manually screened to identify any additional eligible records that may not have been captured through the electronic search.

### Search strategy

The search strategy combined controlled vocabulary terms and free-text keywords related to gastrointestinal endoscopy, procedural sedation, propofol, ketamine, and fentanyl. The search was adapted appropriately for each database. No restriction was placed in terms of language and geographical region. The full search strategies for all databases should be provided in the Supplementary Appendix.

### Selection process

All records retrieved from the database searches were imported into a reference management software program, and duplicates were removed. Two reviewers independently screened the titles and abstracts of all identified records for potential eligibility. Full texts of potentially relevant studies were then retrieved and assessed independently by the same two reviewers against the predefined inclusion and exclusion criteria. Disagreements at any stage were resolved through discussion and consensus; if required, a third reviewer was consulted to reach a final decision.

### Data collection process

Data was extracted independently by two reviewers using standardized and pilot tested data extraction form. Extracted information was again cross-checked for accuracy and completeness. Any discrepancies were resolved through discussion or in consultation with third reviewer. If multiple publications reported findings from same study, data were collated and treated as single study to avoid duplication.

### Risk of bias assessment

Risk of bias assessment was performed independently by two reviewers with disagreements resolved through discussion and consensus. Risk of bias for randomized trials was assessed using Cochrane Risk of Bias 2 (RoB 2) tool (12). Following domains were evaluated: randomization process, deviation from intended intervention, missing outcome data, measurement of outcome, and selection of reported result. Each domain was rated as low risk, some concerns, or high risk of bias and overall study-level judgment was assigned. For non-randomized studies, risk of bias was assessed using ROBINS-I tool (13). These studies would be evaluated across domains of confounding, selection of participants, classification of intervention, deviation from intended intervention, missing data, measurement of outcome, and selection of reported result.

### Synthesis methods

Because substantial heterogeneity was anticipated across included studies in terms of several factors including study population, age group, endoscopic procedure type, sedation depth, drug dosing schedules, other cointerventions, outcome definitions, and timing of outcome assessment, findings were synthesized using narrative synthesis following Synthesis Without Meta-analysis (SWiM) reporting guidelines (14). No quantitative analysis was done.

Narrative synthesis was structured for each of the outcomes by tabulating findings from individual studies and summarized descriptively according to direction and consistency of effect. Dichotomous outcomes like additional propofol requirement and oxygen desaturation were summarized using event count, proportions, and statistical significance wherever available. Continuous outcomes like total propofol dose, systolic blood pressure, mean arterial pressure, and time to recovery were summarized using reported mean with standard deviation or median with interquartile range or other summary statistics as presented.

Synthesis was organized under predefined outcomes of interest. Within each outcome domain, studies were compared in terms of whether findings favoured propofol-ketamine, favoured propofol-fentanyl, or showed no meaningful difference. Narrative interpretation described patterns in evidence rather than vote counting alone and considered study design, sample size, and risk of bias in contextualizing findings.

## Results

The database search identified 1,552 records, of which 333 duplicates were removed before screening. After title and abstract screening of 1,219 records, 87 full-text reports were assessed for eligibility. Among the 66 full-text reports excluded, the most common reasons were different participant populations (*n* = 47), different intervention or comparator (*n* = 16), and lack of relevant outcome data (*n* = 3). Finally, 21 studies were finally included in the review (Figure 1) (6, 8, 9, 15–32).

**Figure 1:**
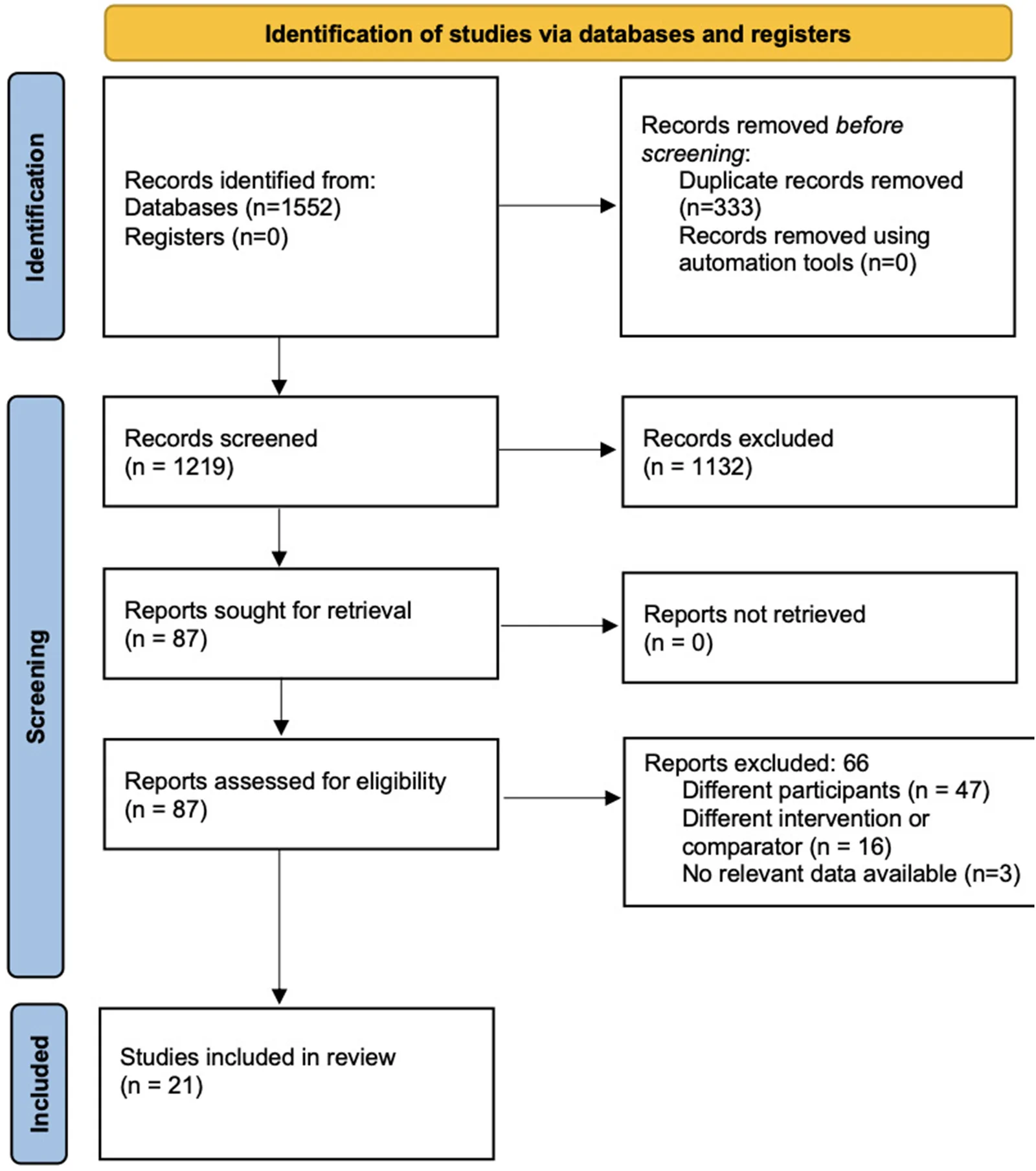
PRISMA flowchart.

### Characteristics of the included studies

Table 1 summarizes the characteristics of the 21 included studies, which were conducted across a wide range of countries, most commonly India, Iran, Egypt, and Turkey, with additional studies from Poland, Brazil, Pakistan, Thailand, Bosnia and Herzegovina, and the United Arab Emirates. The majority of included studies were randomized controlled trials, with only a small number of prospective or retrospective comparative studies. Each of the studies evaluated different gastrointestinal endoscopic procedures like ERCP, colonoscopy, upper GI endoscopy, EGD and PEG indicating broad representation of procedures applied across both diagnostic and therapeutic settings.

**Table 1:** Characteristics of included studies (*N* = 21).

Study	Country	Journal	Study design	Participants details	Procedure	Total sample size (*n*)	PF sample size	PK sample size	Age of PF mean (SD) in years	Age of PK (mean (SD) in years)
Akhondzadeh et al. (15)	Iran	Biomedical journal	RCT	Patients between 18 and 65 years old, who were referred to hospital during 2013–2014 to perform the endoscopy	ERCP	98	49	49	42.22 (7.10)	41.18 (5.98)
Chandar et al. (16)	India	Journal of Pediatric Gastroenterology and Nutrition	RCT	Patients between the age 3 and 12 years	EGD	92	45	47	7.5 (1.4)	8 (1.5)
Chudziński et al. (9)	Poland	Medical Science Monitor	RCT	Patients with a diagnosis of Crohn Disease who were undergoing an elective colonoscopy	Colonoscopy	94	47	47	49 (6.75)	37 (4.75)
Duran et al. (17)	Turkey	Nigerian Journal of Clinical Practice	Retrospective study	patients who underwent Percutaneous Endoscopic Gastrostomy in the Endoscopy Unit	PEG	71	40	31	60.7 (26.37)	69.26 (18.57)
Falcão et al. (6)	Brazil	Brazilian Journal of Anesthesiology	RCT	Patients aged 18 to 65 years, and scheduled for elective Colonoscopy or bidirectional endoscopy	Colonoscopy and upper endoscopy	80	40	40	52.0 (12.3)	53.7 (9.8)
Gad EL-Rab et al. (18)	Egypt	Journal of Current Medical Research and Practice	RCT	Children aged 6–12 years, who were scheduled for elective endoscopy	UGIE	60	30	30	9.27 (2.13)	8.90 (2.35)
Gorji et al. (19)	Iran	Anesthesia and Pain Medicine	RCT	Patients aged 30–70 years old, who undergone endoscopy	ERCP	72	42	30	60.50 (15.66)	56 (19.75)
Hasanein and El-Sayed (20)	Egypt	Egyptian Journal of Anaesthesia	RCT	Adults aged from 18 to 70 years, who are obese with BMI 25–35	ERCP	200	100	100	56.93 (11.9)	57.67 (13.3)
Javaid et al. (21)	Pakistan	Pakistan Journal of Medical and Health Sciences	RCT	Only ASA I and II patients with a BMI of less than or equal to 25 kg/m2 and aged 18 to 50 years scheduled for ERCP	ERCP	124	62	62	38.68 (12.17)	38.90 (13.10)
Kamel et al. (22)	Egypt	Journal of Anesthesia & Clinical Research	RCT	Patients scheduled for diagnostic upper endoscopy	Upper GI EUS	90	45	45	35.71 (14.92)	41.6 (18.01)
Khajavi et al. (23)	Iran	Anesthesia and Pain Medicine	RCT	Patients older than 18 enrolled for the procedure	Colonoscopy	60	30	30	51.60 (21)	55.9 (15)
Kovačević et al. (8)	Bosnia and Herzegovina	Saudi Journal of Anesthesia	RCT	Patients who were older than 18 years	Colonoscopy	60	30	30	56.17 (12.24)	57.47 (12.10)
Kumar et al. (24)	India	International Journal of Current Pharmaceutical Review and Research	Prospective study	Adult patients, aged 18–65 years, scheduled for elective ERCP	ERCP	100	50	50	53.1 (10.6)	52.8 (11.2)
Pathanon et al. (25)	Thailand	Journal of the Department of Medical Services	RCT	Patients aged 18–85 years	Colonoscopy	64	32	32	59.56 (11.43)	59.53 (8.90)
Prem et al. (26)	India	Bali Journal of Anesthesiology	RCT	Adults aged 18–70 years of either sex with body mass index of >30 kg/m^2^	ERCP	100	50	50	51.88 (11.00)	50.38 (11.80)
Reddy and Gupta (27)	India	International Journal of Medical Anesthesiology	RCT	Patients aged of 18–60 years, scheduled for endoscopy	ERCP	60	30	30	NR	NR
Safadi et al. (28)	United Arab Emirates	Journal of Neonatal Surgery	Prospective study	Patients aged 18–60 years, with an ASA physical status of 1–4	Endoscopic Procedures	100	50	50	29	29
Seleem et al. (29)	Egypt	Current Drug Safety	RCT	Adult patients who were referred for the endoscopy	Colonoscopy	150	75	75	NR	NR
Singh et al. (30)	India	Korean Journal of Anesthesiology	RCT	Adult aged 18–60 years of either gender who scheduled for abdominal EUS and FNAC by a single endoscopist	Upper GI EUS	142	70	72	49.3 (14.5)	44.4 (16.4)
Takzare et al. (31)	Iran	Archives of Anesthesiology and Critical Care	RCT	Children aged 2 to 12 years, with an ASA physical status of I or II	UGIE	130	65	65	8.1	8.2
Tosun et al. (32)	Turkey	Pediatric Anesthesia	RCT	Children between the ages of 1 and 16	UGIE	90	44	46	11.25 (3.9)	9.66 (4.87)

ASA, American Society of Anesthesiologists; BMI, body mass index; EGD, esophagogastroduodenoscopy; ERCP, endoscopic retrograde cholangiopancreatography; EUS, endoscopic ultrasound; FNAC, fine needle aspiration cytology; NR, not reported; PEG, percutaneous endoscopic gastrostomy; PF, propofol and fentanyl group; PK, propofol and ketamine group; RCT, randomized controlled trial; SD, standard deviation; UGIE, upper gastrointestinal endoscopy.

Overall, included participants were heterogeneous, containing both paediatric and adult patients. Sample sizes ranged from 60 to 200 participants. Most studies had balanced group allocation between propofol-fentanyl and propofol-ketamine arm. Participant ages varied substantially based on study population and procedure type as several paediatric studies involved upper gastrointestinal endoscopy and several adult studies involved ERCP or colonoscopy.

### Baseline clinical profile and sedation regimens amongst included studies

Table 2 presents baseline clinical profile of participants and the sedation regimens used across included studies. Most included studies had patients belonging to ASA class I-III with fewer studies enrolling higher-risk populations like ASA class III–IV or ASA class I–IV patients. Wherever reported, body weight was comparable between propofol-fentanyl and propofol-ketamine groups within individual studies. With regard to sedation protocol, propofol was most commonly administered at doses ranging from 0.5 to 1.2 mg/kg, while fentanyl was usually given at 1 mcg/kg. Ketamine was most commonly administered at 0.5 mg/kg, while some studies used lower doses like 0.25 mg/kg or higher doses up to 1 mg/kg.

**Table 2:** Baseline clinical characteristics and sedation drug doses used in the included studies (*N* = 21).

Study	ASA class	Weight PF (kg)	Weight PK (kg)	Male PF (%)	Male PK (%)	Propofol dose	Fentanyl dose	Ketamine dose
Akhondzadeh et al. (15)	III and IV	66.09 (7.12)	67.94 (6.24)	NR	NR	0.5 mg/kg	1 mcg/kg	0.5 mg/kg
Chandar et al. (16)	I to III	19.05 (2.75)	18.89 (2.45)	60	51.1	0.5 mg/kg	1 mcg/kg	0.5 mg/kg
Chudziński et al. (9)	I and II	NR	NR	40.8	46.8	0.5 mg/kg	1 mcg/kg	0.5 mg/kg
Duran et al. (17)	III and IV	NR	NR	52.5	54.8	0.5–1.2 mg/kg	0.2–1 mcg/kg	0.2–0.6 mg/kg
Falcão et al. (6)	I and II	NR	NR	25	37.5	1 mg/kg	0.25 mcg/kg	0.25 mg/kg
Gad EL-Rab et al. (18)	I and II	24.7 (3.96)	24.03 (5.05)	43.3	50	1.5 mg/kg	1 mcg/kg	0.5 mg/kg
Gorji et al. (19)	III and IV	NR	NR	47.62	43.33	0.5 mg/kg	50–100 mcg	0.5 mg/kg
Hasanein and El-Sayed (20)	I to III	NR	NR	50	49	0.5 mg/kg	NR	NR
Javaid et al. (21)	NR	NR	NR	48.39	35.48	0.5 mg/kg	1 mcg/kg	0.5 mg/kg
Kamel et al. (22)	I to III	75.16 (15.92)	78.09 (9.64)	64.4	46.7	1 mg/kg	1 mcg/kg	0.25 mg/kg
Khajavi et al. (23)	I to III	56 (14)	59 (17)	60	53.33	0.5 mg/kg	1 mcg/kg	0.5 mg/kg
Kovačević et al. (8)	I and II	76.27 (12.18)	76.03 (12.17)	26.67	36.67	0.5 mg/kg	1 mcg/kg	0.5 mg/kg
Kumar et al. (24)	I and II	64.3 (8.4)	65.7 (7.9)	56	60	NR	NR	NR
Pathanon et al. (25)	I to III	61.56 (14.01)	59.78 (11.58)	34.4	28.1	1 mg/kg	1 mcg/kg	0.5 mg/kg
Prem et al. (26)	I and II	55.60 (11.49)	56.72 (9.54)	50	48	1 mg/kg	1 mcg/kg	0.5 mg/kg
Reddy and Gupta (27)	I and II	NR	NR	46.67	43.33	0.5 mg/kg	1 mcg/kg	0.5 mg/kg
Safadi et al. (28)	I to IV	NR	NR	NR	NR	1–2 mg/kg	1 mcg/kg	0.5 mg/kg
Seleem et al. (29)	NR	NR	NR	49.33	53.33	1 mg/kg	1 mcg/kg	0.5 mg/kg
Singh et al. (30)	I and II	NR	NR	55.71	63.89	NR	50 mcg	0.5 mg/kg
Takzare et al. (31)	I and II	22.1 (1.86)	22.35 (1.02)	76.9	76.9	1.2 mg/kg	1 mcg/kg	1 mg/kg
Tosun et al. (32)	I and II	36.7 (14.2)	30.2 (16.2)	54.5	47.8	1.2 mg/kg	1 mcg/kg	1 mg/kg

kg, kilogram; mg, milligram; mcg, microgram; NR, not reported.

### Risk of bias assessment

Risk-of-bias assessment showed variable methodological quality across the included studies. Among the randomized controlled trials (Figure 2), five studies were judged to be at low risk of bias overall, while rest of the RCTs had either some concerns or high risk of bias, most commonly due to concerns in the randomization process, missing outcome data, and measurement of outcomes. High overall risk of bias in seven trials was largely driven by shortcomings in one or more individual RoB 2 domains, particularly bias due to missing outcome data and outcome measurement.

**Figure 2:**
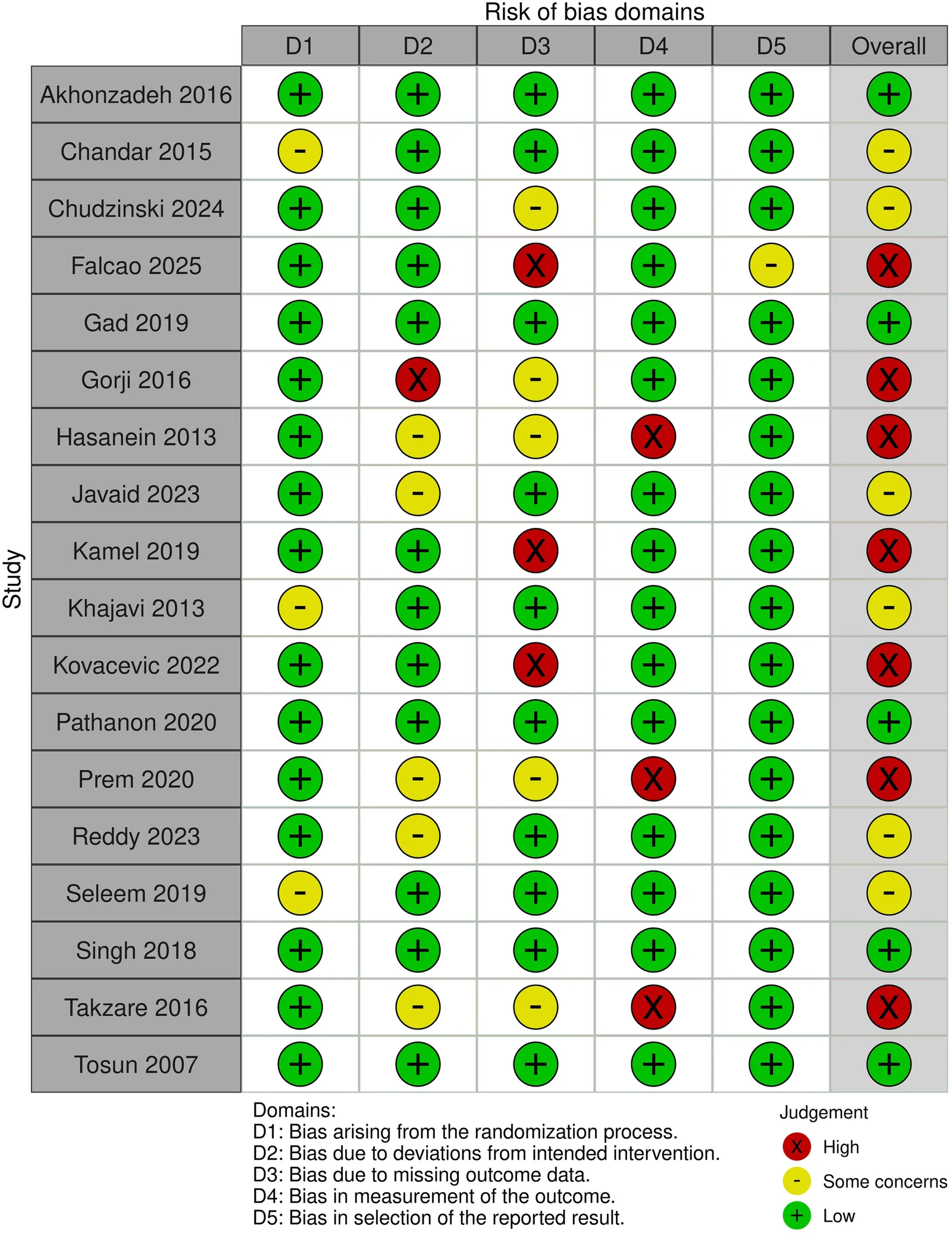
Risk of bias assessment amongst the included randomized controlled trials.

Among the non-randomized comparative studies (Figure 3), one study was judged to be at low risk of bias overall, one had moderate risk of bias, and one was judged to have critical risk of bias. The critical risk rating was mainly attributable to bias in participant selection, while moderate concerns were noted in some studies for confounding, classification of interventions, missing data, and selective reporting. Overall, these findings indicate that although several studies were methodologically robust, the certainty of the evidence is tempered by important risk-of-bias concerns in a number of included studies.

**Figure 3:**
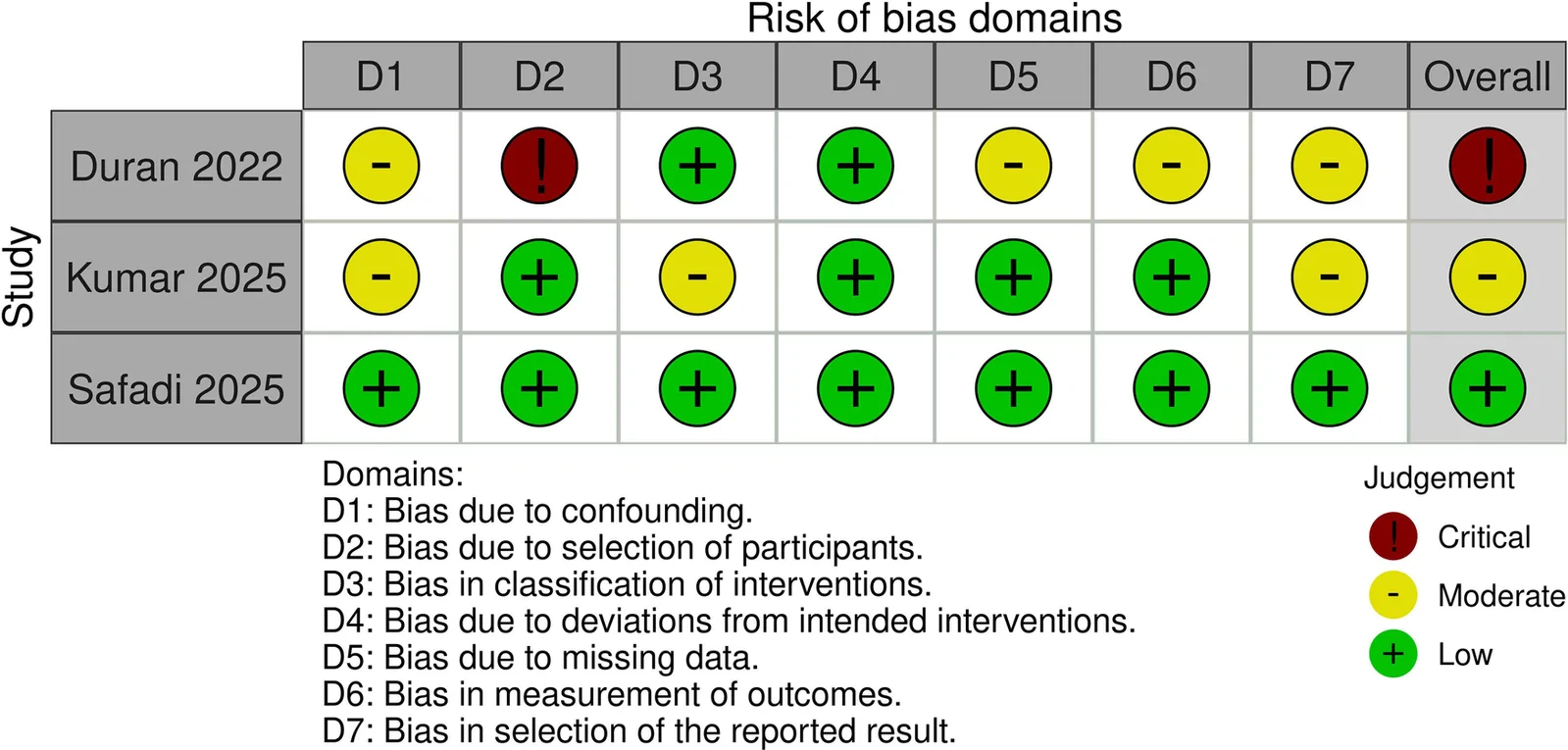
Risk of bias assessment amongst the included non-randomized controlled trials.

### Narrative synthesis of outcomes

Because substantial heterogeneity was observed across studies in terms of procedure type, participant characteristics, sedation protocols, outcome definitions, and timing of outcome assessment, the findings were synthesized narratively and are presented outcome-wise in Tables 3–8. Across the included studies, the most consistent patterns favoured propofol–ketamine for reduced additional propofol requirement, lower oxygen desaturation, lower total propofol consumption, and better preservation of blood pressure, whereas findings for recovery time were more variable.

**Table 3:** Narrative summary of studies reporting requirement for additional propofol doses in the first minute.

Study ID	Outcome
Chandar et al. (16)	40% (18/45) of patients in the PF group required additional doses compared to 21.3% (10/47) in the PK group.
Duran et al. (17)	12 patients in Group PF and zero patients in Group PK, required additional doses of propofol.
Gad EL-Rab et al. (18)	The number of patients requiring additional propofol was not significantly different, occurring in 50% (14/30) of the PF group and 46.6% (15/30) of the PK group
Tosun et al. (32)	Significantly fewer patients in the PK group (17%) required additional propofol in the first minute compared to the PF group (50%).

**Table 4:** Narrative summary of studies reporting oxygen desaturation.

Study ID	Outcome
Chandar et al. (16)	Minor desaturation (75%–90% for <60s) and sentinel desaturation (<90% for >60s or <75% at any time) occurred in 8.9% of the PF group and 6.5% of the PK group (*P* = 0.714)
Chudziński et al. (9)	Desaturation events were significantly more common in the PF group (42.6%) than in the KET (6.4%) or LID (7.7%) groups (*P* < 0.001)
Duran et al. (17)	Desaturation was observed in 22.5% (9 patients) of Group PF compared to only 9.6% (3 patients) of Group PK (*P* = 0.023)
Kamel et al. (22)	Oxygen desaturation was recorded in 13.3% of Group PK and 8.9% of Group PF
Kumar et al. (24)	Oxygen saturation drops below 90% were significantly more common in the PF group (42.6%) than in the PK (6.4%)
Reddy and Gupta (27)	There was no statistically significant difference in desaturation requiring assisted ventilation between the KP group (3.30%) and the FP group (6.70%) with a *p*-value of 0.554
Tosun et al. (32)	Mean oxygen saturation levels were statistically comparable between the two groups during the procedure, and transient hypoxia (<90%) occurred in similar numbers (PK: 3, PF: 4)
Hasanein and El-Sayed (20)	A significant difference was observed in oxygen desaturation, which occurred in 7% of patients in the FP group and 0% in the KP group (*p* = 0.021)
Kovačević et al. (8)	The incidence of desaturation (SpO2 < 95%) was significantly higher in the FP group 19 patients (63.3%) compared to the PK group 15 patients (50.0%) (*p* = 0.033).
Prem et al. (26)	The incidence of desaturation (SpO2 < 90%) was higher in the PF group (16%) than in the PK group (4%), this difference was not statistically significant
Singh et al. (30)	The incidence of minor desaturation (SpO2 < 95%) was statistically similar across the groups, occurring in 7.35% of Group PF, 5.71% of Group PK.

**Table 5:** Narrative summary of studies reporting total propofol dose.

Study ID	Outcome
Chandar et al. (16)	Propofol used from the initial mandatory dose to the end of the procedure was a median of 7 doses for both the PF (IQR 6–9.5) and PK (IQR 6–9) groups
Chudziński et al. (9)	FNT group used significantly more (0.14 mg/kg/min) than either the KET group (0.10 mg/kg/min, *P* < 0.001) or the LID group (0.12 mg/kg/min, *P* = 0.001)
Duran et al. (17)	Amount of propofol required for the procedure, which was significantly higher in Group PF (64.37 ± 25.9 mg) compared to Group PK (34.8 ± 11.7 mg) with a *p*-value of <0.001
Kumar et al. (24)	Patients in the fentanyl (PF) group required a significantly higher total amount of propofol (mean 136.81 mg) than those in the ketamine (PK) group (mean 108.3 mg, *P* = 0.017)
Reddy and Gupta (27)	The total amount of propofol consumed was significantly higher in the FP group (109.883 ± 11.3871 mg) compared to the KP group (89.867 ± 9.8942 mg), yielding a highly significant *p*-value of <0.001
Akhondzadeh et al. (15)	The total dose of propofol used was statistically similar between the PK group (148.67 ± 71.24 mg) and the PF group (134.42 ± 63.66 mg), with a non-significant *p*-value of 0.36
Hasanein and El-Sayed (20)	The total dose of propofol required was significantly lower in the KP group (57.71 ± 16.97 mg) compared to the FP group (97.08 ± 23.31 mg), yielding a highly significant *p*-value of <0.01
Prem et al. (26)	The total dose of propofol required was significantly higher in the PF group (176.9 ± 50.28 mg) than in the PK group (153.7 ± 57.16 mg), resulting in a statistically significant *p*-value of 0.033
Singh et al. (30)	The median amount of propofol consumed was significantly higher in Group PF (9.25 [7.3–13.2] mg/kg/h) compared to Group PK (8.8 [6.8–12.2] mg/kg/h, *p* = 0.024)

**Table 6:** Narrative summary of studies reporting systolic blood pressure.

Study ID	Outcome
Kumar et al. (24)	The KET group experienced a significantly smaller drop in systolic pressure (−7.19 mmHg) compared to both the FNT (−16.74 mmHg) and LID (−13.19 mmHg) groups (*P* < 0.001)
Pathanon et al. (25)	Patients in the ketofol group had significantly higher SBP (132 ± 13.48 mmHg) compared to the fenofol group (124 ± 15.83 mmHg), yielding a *p*-value of 0.025
Reddy and Gupta (27)	During the procedure, patients in the ketofol group had significantly higher SBP (132 ± 13.48 mmHg) compared to the fenofol group (124 ± 15.83 mmHg), yielding a *p*-value of 0.025
Gad EL-Rab et al. (18)	Mean systolic blood pressure was significantly lower in the PF group compared to the PK group at the end (95.20 ± 7.31 vs. 99.10 ± 7.88 mmHg, *p* = 0.030)
Safadi et al. (28)	The PK group maintained significantly higher and more stable systolic blood pressure compared to the PF group at END (119.4 ± 13.085 vs. 107.2 ± 13.368 mmHg; *p* = 0.001)
Takzare et al. (31)	The mean systolic blood pressure during the procedure was significantly lower in the PF group (92.12 ± 1.55 mmHg) than in the PK group (93.22 ± 2.90 mmHg), with a *p*-value of 0.026
Akhondzadeh et al. (15)	Mean systolic blood pressure remained statistically comparable between the two groups throughout the monitoring period, including at the 20-minute mark (PK: 135.94 ± 18.57 vs. PF: 139.84 ± 18.57 mmHg; *p* = 0.30)

**Table 7:** Narrative summary of studies reporting time to recovery.

Study ID	Outcome
Chandar et al. (16)	The duration required to reach a modified Aldrete scale score of 9 or higher was not significantly different, with a median of 26 min for the PF group and 29.5 min for the PK group
Duran et al. (17)	Time from the end of the procedure until the Aldrete score reached 8, this was significantly longer in Group K (10.87 ± 2.75 min) than in Group F (8.25 ± 2.7 min), yielding a *p*-value of <0.001
Kumar et al. (24)	The interval until an Aldrete score of 8 was reached was significantly longer in Group K (10.87 min) than in Group F (8.25 min, *P* < 0.001)
Reddy and Gupta (27)	Time to reach an acceptable recovery score based on Modified Aldrete Score was significantly faster in the FP group (9.8 ± 0.407 at 30 min) compared to the KP group (9.5 ± 0.509; *p* = 0.015), likely because Ketamine takes longer to clear from the body
Falcão et al. (6)	The median time to reach a Ramsay Sedation Scale (RSS) score of 2 was identical for both groups at 10 min, showing no statistical difference
Gorji et al. (19)	The mean time to reach a recovery on Aldrete score of >9 was statistically similar between the PK group (14.17 ± 4.564 min) and the PF group (12.86 ± 3.339 min) with a *p*-value of 0.164
Javaid et al. (21)	The mean Recovery time in minute for PF group is 14.40 ± 2.03 and PK group is 14.14 ± 1.93, yielding a *p*-value of 0.47
Khajavi et al. (23)	The mean recovery time was 15.6 ± 6.2 min for the PK group compared to 12.3 ± 8.4 min for the PF group
Prem et al. (26)	The mean time to reach a discharge-ready Aldrete score of ≥9 was significantly longer in the PK group (11.12 ± 2.75 min) compared to the PF group (9.72 ± 3.02 min), with a *p*-value of 0.017

**Table 8:** Narrative summary of studies reporting mean arterial pressure.

Study ID	Outcome
Chudziński et al. (9)	The absolute decrease in MAP from baseline was significantly milder in the KET group (−5.47 mmHg) than in the FNT group (−9.89 mmHg, *P* = 0.0416)
Kumar et al. (24)	The PK group maintained a significantly higher mean arterial pressure (80.9 mmHg) compared to the PF group (77.8 mmHg) specifically at the 8-minute mark (*P* = 0.021)
Pathanon et al. (25)	ketofol group maintained a significantly higher MAP (93.03 ± 10.32 mmHg) than the fenofol group (86.75 ± 11.51 mmHg), with a statistically significant difference of *P* = 0.025
Safadi et al. (28)	The Ketofol group exhibited significantly higher mean arterial pressure readings throughout key intraoperative points, specifically at 6 min (89.34 ± 11.974 vs. 84.2 ± 12.172 mmHg; *p* = 0.019), 9 min (88.89 ± 11.134 vs. 83.56 ± 11.465 mmHg; *p* = 0.041), and 15 min (90.38 ± 13.48 vs. 81.8 ± 10.729 mmHg; *p* = 0.024)
Seleem et al. (29)	The Propofol-Ketamine group demonstrated significantly higher Mean Arterial Pressure during (93.9 ± 10.9 vs. 85.45 ± 6.7 mmHg; *p* = 0.03) and after the procedure (94.8 ± 10.8 vs. 87.2 ± 6.8 mmHg; *p* = 0.042) compared to the Propofol-Fentanyl group

### Requirement for additional propofol doses in the first minute

Four studies reported the requirement for additional propofol doses during the first minute of the procedure (Table 3). Overall, the findings generally favored the propofol–ketamine group, with most studies showing that patients receiving propofol–ketamine required fewer supplemental propofol doses than those receiving propofol–fentanyl. Chandar et al. (16) reported a lower proportion of patients requiring additional propofol in the PK group than in the PF group, and Tosun et al. (32) similarly found a markedly lower need for rescue propofol with PK. Duran et al. (17) also reported that additional propofol was required in patients from the PF group but not in the propofol–ketamine group. In contrast, Gad EL-Rab et al. (18) found no significant difference between the two groups, with both regimens showing a similar frequency of additional propofol requirement. Taken together, the narrative evidence suggests that propofol–ketamine may reduce the need for early supplemental propofol, although this finding was not uniform across all studies.

### Oxygen desaturation

Oxygen desaturation was one of the most frequently reported safety outcomes and was described in 11 studies (Table 4). Across these studies, the overall pattern generally favoured propofol-ketamine, with several studies reporting fewer desaturation events in the propofol–ketamine group than in the propofol-fentanyl group. This trend was particularly evident in Chudziński et al. (9), Duran et al. (17), Kumar et al. (24), Hasanein and El-Sayed (20), and Kovačević et al. (8), all of which reported higher desaturation rates in the fentanyl-containing regimen. Prem et al. (26) also observed fewer desaturation events in the PK group, although the difference was not statistically significant. However, fewer studies showed direction of effect in different manner. Kamel et al. (22) reported slightly more desaturation in propofol-ketamine group, while Chandar et al. (16), Reddy and Gupta (27), Tosun et al. (32), and Singh et al. (30) found no clear difference between two regimens. Interpretation of this outcome requires caution as definition of desaturation varied across studies, ranging from SpO2 thresholds below 90% or 95% to composite definitions incorporating duration and severity. Despite this heterogeneity, the overall narrative pattern suggests that propofol-ketamine was mostly associated with lower rate of oxygen desaturation than propofol-fentanyl.

### Total propofol dose

Nine studies reported total propofol consumption, and most studies have reported that there was lower propofol requirement with propofol-ketamine compared to propofol-fentanyl (Table 5). Studies like Chudziński et al. (9), Duran et al. (17), Kumar et al. (24), Reddy and Gupta (27), Hasanein and El-Sayed (20), Prem et al. (26), and Singh et al. (30) reported high propofol use in fentanyl group, indicating propofol sparing effect with ketamine. However, Chandar et al. (16) study found similar propofol requirements, and Akhondzadeh et al. (15) reported no statistically significant difference between both groups. Nevertheless, narrative synthesis indicates that addition of ketamine to propofol mostly reduced total propofol consumption compared with fentanyl.

### Systolic blood pressure

Seven studies reported systolic blood pressure (Table 6). Most of these studies found that propofol-ketamine regimen was associated with higher or more stable systolic blood pressure during procedure compared with propofol-fentanyl. Kumar et al. (24) reported smaller reduction in systolic blood pressure in ketamine group, while studies like Pathanon et al. (25), Reddy and Gupta (27), Gad EL-Rab et al. (18), Safadi et al. (28), and Takzare et al. (31) found significantly higher systolic blood pressure values in propofol-ketamine group at intra-procedural or end-procedural time points. Only Akhondzadeh et al. (15) reported similar systolic blood pressure values between both groups. Overall, despite variation in time points used for assessment, narrative evidence supports better hemodynamic preservation with propofol ketamine combination than propofol-fentanyl combination.

### Time to recovery

Eight studies reported recovery time, and findings were mixed than respiratory and hemodynamic outcomes (Table 7). Several studies like Duran et al. (17), Kumar et al. (24), Reddy and Gupta (27), Khajavi et al. (23), and Prem et al. (26) suggested longer recovery period with propofol-ketamine than propofol-fentanyl. In contrast, Chandar et al. (16), Falcão et al. (6), and Gorji et al. (19) found no difference in recovery time between two groups. Recovery was defined using different methods with some studies using Aldrete or Modified Aldrete score while other studies using Ramsay Sedation Scale threshold.

Recovery time was reported across the included studies, with considerable variability between propofol–ketamine and propofol–fentanyl regimens. Several studies reported longer recovery or discharge times with propofol–ketamine, whereas others found no significant difference between the two groups. A small number of studies reported faster recovery or shorter discharge times with propofol–ketamine. Overall, the findings were inconsistent, although longer recovery was observed more frequently with ketamine-containing regimens. Differences in ketamine dose, sedation protocols, recovery criteria, and timing of assessment may partly explain this variability.

### Mean arterial pressure

Five studies reported mean arterial pressure, and all of them favoured propofol-ketamine group (Table 8). Chudziński et al. (9) found slight decline in mean arterial pressure from baseline with ketamine combination, and studies like Kumar et al. (24), Pathanon et al. (25), Safadi et al. (28), and Seleem et al. (29) reported significantly higher mean arterial pressure values in propofol ketamine combination group during intraprocedural or postprocedural time points. Although exact timing of measurement differed across studies, direction of effect was consistent, indicating that propofol-ketamine provided better maintenance of mean arterial pressure during endoscopic sedation.

### Reported adverse events

Adverse events were variably reported across the included studies. Overall, propofol–ketamine was generally associated with fewer respiratory and hemodynamic adverse events, including apnea, hypoxia or desaturation, hypotension, and bradycardia, compared with propofol–fentanyl in several studies. Some studies also reported fewer requirements for airway intervention with propofol–ketamine (Table 9). However, ketamine-containing regimens were associated with certain adverse effects, particularly hallucinations, emergence reactions, dizziness, and, in some pediatric populations, nausea, vomiting, or airway-related events. The frequency and definitions of adverse events varied considerably between studies, limiting direct comparison. Overall, the findings suggest a possible reduction in respiratory and hemodynamic complications with propofol–ketamine, while ketamine-related psychomimetic and gastrointestinal adverse effects should also be considered.

**Table 9:** Adverse events reported with propofol–ketamine versus propofol–fentanyl sedation.

Study	Patient population	Reported adverse events (PK vs. PF/others)
Akhondzadeh et al. (15)	Adults	Apnea: 32% PK vs. 63% PF. No laryngospasm observed in either group.
Chandar et al. (16)	Pediatric	Propofol injection pain: 31.9% PK vs. 60% PF. Sentinel desaturation: 6.5% PK vs. 8.9% PF. BP decrease ≥25%: 14.9% PK vs. 31.1% PF.
Chudziński et al. (9)	Crohn’s patients	Desaturation: 6.4% PK vs. 42.6% PF. Dizziness: 17.0% PK vs. 19.1% PF. Metallic taste: 13.5% (Lidocaine group) vs. 2.1% (PF/PK).
Duran et al. (17)	Adults	Desaturation: 9.6% PK vs. 22.5% PF. Hypotension: 0% PK vs. 10% PF.
Falcão et al. (6)	Adults	Cardiovascular complications: 37.5% PK vs. 62.5% PF.
Gad EL-Rab et al. (18)	Pediatric	Hypoxia (transient): 0% PK vs. 10% PF. Nausea/Vomiting: 3.3% PK vs. 6.6% PF.
Gorji et al. (19)	Adults	Apnea: 3.3% PK vs. 16.7% PF. Nausea/Vomiting: 1 case (PK) vs. 4 cases (PF).
Hasanein and El-Sayed (20)	Obese	Apnea: 2% PK vs. 10% PF. Hypotension: 3% PK vs. 12% PF. Bradycardia: 1% PK vs. 9% PF. PONV: 3% PK vs. 1% PF.
Javaid et al. (21)	Adults	Apnea: 5 patients (PK) vs. 7 patients (PF).
Kamel et al. (22)	Adults	Unstable respiration: 13.3% PK vs. 8.9% PF. Hallucination: 6.7% PK vs. 0% PF.
Khajavi et al. (23)	Adults	Nausea/Vomiting: 12.5% in both groups. Emergence reactions: 7.5% PK vs. 0% PF.
Kovačević et al. (8)	Adults	Desaturation: 50% PK vs. 63.3% PF. Weakness: 50% PK vs. 66.7% PF. Hallucinations: 6.7% PK vs. 0% PF.
Kumar et al. (24)	Adults	Apnea: 3.3% PK vs. 16.7% PF. Intubation required: 0% PK vs. 6% PF. Nausea/Vomiting: 2% PK vs. 8% PF.
Pathanon et al. (25)	Adults	Airway management: 9.4% PK vs. 31.2% PF. Hallucination/Nightmares: 12.5% PK vs. 0% PF.
Prem et al. (26)	Adults	Hypotension: 6% PK vs. 24% PF. Bradycardia: 2% PK vs. 18% PF. Emergence reaction: 10% PK vs. 0% PF.
Reddy and Gupta (27)	Adults	Bradycardia (req. Atropine): 0% PK vs. 13.3% PF. Hypotension (req. Fluid): 0% PK vs. 10% PF.
Safadi et al. (28)	Adults	Regimens provided adequate anesthesia without serious adverse events.
Seleem et al. (29)	Adults	Hypoxia: PK (3/75) vs. PF (12/75). PONV: PK (8/75) vs. PF (28/75). Hallucinations: PK (10/75) vs. PF (2/75).
Singh et al. (30)	Adults	Hiccups: 41.67% PK vs. 40.0% PF vs. 35.2% (Propofol-only). Coughing: 9.72% PK vs. 7.14% PF.
Takzare et al. (31)	Pediatric	Nausea/Vomiting: 53.8% PK vs. 32.3% PF. Bronchospasm/Laryngospasm: 9% PK vs. 2% PF.
Tosun et al. (32)	Pediatric	Dizziness: PK (15 pts) vs. PF (4 pts). Vomiting: PK (7 pts) vs. PF (0 pts). Restlessness: PK (2 pts) vs. PF (10 pts).

## Discussion

This review provides focused comparison of propofol-ketamine and propofol-fentanyl sedation strategies for gastrointestinal endoscopic procedures and shows consistent overall pattern despite heterogeneity across procedures, populations, drug doses, and outcome definitions. Clearest signal across included studies was that propofol-ketamine was associated with lower supplemental propofol requirements, lower total propofol consumption, and better preservation of hemodynamic parameters, particularly systolic blood pressure and mean arterial pressure. Another important finding was tendency toward fewer oxygen desaturation events with propofol-ketamine in majority of studies, although this outcome was less uniform because desaturation thresholds and reporting formats varied considerably. In contrast, recovery related findings were more mixed, with several studies suggesting longer recovery after ketamine containing regimens, while others found little or no difference.

The findings indicate that propofol–ketamine generally reduces supplemental and total propofol requirements and is associated with better preservation of respiratory and hemodynamic parameters across many studies. In contrast, recovery outcomes were less consistent, with several studies reporting longer recovery with ketamine-containing regimens. Overall, these findings suggest that propofol–ketamine may offer advantages in intraprocedural stability and propofol sparing, while the choice of adjunct should remain individualized according to patient characteristics, procedural requirements, and the relative importance of recovery time.

Several factors may influence the observed outcomes of sedation and may partly explain the variability between studies. Patient characteristics, particularly age, baseline cardiovascular status, ASA physical status, body habitus, and the presence of comorbidities, can modify both the pharmacodynamic response to sedative drugs and the risk of respiratory or hemodynamic adverse events. The included studies enrolled heterogeneous populations, ranging from pediatric patients to older adults and from relatively low-risk ASA I–II populations to patients with ASA III–IV status. Procedural characteristics may also be important, as the included studies evaluated different procedures, including colonoscopy, upper gastrointestinal endoscopy, ERCP, endoscopic ultrasound, and PEG, which differ in procedural stimulation, duration, airway interference, and analgesic requirements. In addition, differences in propofol, fentanyl, and ketamine doses, drug administration techniques, sedation depth, and use of additional sedative or analgesic agents may influence propofol requirements, oxygenation, blood pressure, and recovery time. Monitoring practices and the criteria used to define outcomes may further contribute to between-study variation. For example, oxygen desaturation was defined using different thresholds and durations, while recovery was assessed using different scoring systems and time points. Therefore, the observed advantage of propofol–ketamine for propofol sparing and hemodynamic or respiratory stability should be interpreted in the context of these patient-, procedure-, protocol-, and outcome-related factors rather than considered a uniform effect across all gastrointestinal endoscopic settings.

Beyond drug selection, the clinical setting and intensity of sedation may substantially modify the safety and recovery profile of propofol-based regimens. Deeper sedation is associated with greater risk of airway obstruction, hypoventilation, and haemodynamic instability, while patient factors such as advanced age, obesity, cardiopulmonary disease, and higher ASA physical status may increase susceptibility to these effects (33, 34). The type and complexity of endoscopic procedure are also relevant because more stimulating or prolonged procedures may require greater analgesic and sedative requirements, potentially increasing cumulative propofol exposure (33). In this context, an analgesic adjunct that reduces propofol requirements may have greater clinical relevance in selected patients or procedures. However, the potential benefits of ketamine should be balanced against its own adverse-effect profile, including psychomimetic reactions, nausea and vomiting, and recovery-related effects, which may become more clinically relevant with increasing dose or prolonged administration (35, 36). These considerations support an individualized approach to sedation rather than assuming that either propofol–ketamine or propofol–fentanyl is universally preferable.

Taken together, these findings suggest that main advantage of propofol-ketamine in endoscopic sedation lies in physiologic stability and propofol sparing effect, whereas main advantage of propofol-fentanyl may be tendency toward shorter or simpler recovery in certain settings (37). In endoscopy, best sedation regimen is rarely the one that optimizes single endpoint. Instead, it is regimen that offers most acceptable compromise between procedural conditions, respiratory safety, cardiovascular stability, and throughput. Our review therefore does not suggest that one regimen is universally superior in every context. Rather, it indicates that propofol-ketamine may be especially attractive when avoiding desaturation, minimizing hypotension, or reducing additional propofol administration is priority, while propofol-fentanyl may still remain as an option when rapid post procedure recovery is emphasized and clinical setting allows careful respiratory monitoring.

Our findings align with broader pharmacologic and clinical literature on procedural sedation, particularly literature suggesting that ketamine containing regimens can offset few of cardiorespiratory problems of propofol based sedation. Recent meta-analysis directly comparing propofol-fentanyl with propofol-ketamine in gastrointestinal endoscopy reported higher rates of desaturation and hypotension in propofol-fentanyl group, while also suggesting that propofol-fentanyl may shorten procedure time and possibly improve recovery time in some circumstances (38). This overall findings is in line with our narrative synthesis, especially regarding respiratory and hemodynamic outcomes, while also supporting finding that recovery may be one of the domains in which fentanyl containing regimens can appear more favourable (39).

Recent endoscopy specific evidences also supports present review findings. Recent systematic review of adjunctive esketamine added to propofol based sedation for gastrointestinal endoscopy found reductions in hypotension, adverse respiratory events, and propofol consumption without prolonging recovery time overall (40). Although esketamine is not identical to racemic ketamine and comparator framework differed from ours, physiologic direction is similar and reinforces idea that NMDA antagonist adjuncts may improve safety profile of propofol based endoscopic sedation by reducing both hemodynamic compromise and propofol dose requirements (40).

Recent reviews of current and emerging sedation practices in endoscopy emphasize that propofol is central to modern endoscopic sedation, but optimal adjunct drug varies according to procedural intensity, setting, and patient risk profile (4). Our review fits well within this paradigm as our findings favour propofol-ketamine on several safety and intraprocedural performance domains. However, they also suggest that selection of regimen should remain tailored rather than universal recommendation for all.

The findings of this review are also biologically plausible. Propofol is rapid onset hypnotic highly effective for procedural sedation, but depending on dose can cause respiratory depression, loss of airway tone, and hypotension through vasodilation and myocardial depression. Ketamine provides analgesia and dissociative sedation while preserving spontaneous respiration and exerting sympathomimetic effects supporting blood pressure and heart rate. When these two drugs are combined, ketamine may partially counterbalance hemodynamic and respiratory depressant effects of propofol, while propofol may temper some of dysphoric or emergence-related effects of ketamine. This interaction mechanism offers strong mechanistic explanation for lower propofol requirements and better blood pressure stability observed across included studies.

Reduced need for add-on propofol dose and lower total propofol consumption with propofol-ketamine reflects both improved analgesia and more stable procedural sedation (41, 42). Fentanyl is effective analgesic but it does not provide same dissociative component as ketamine. During stimulating parts of endoscopic procedure, especially therapeutic procedures or those requiring prolonged manipulation, ketamine may contribute effectively to immobilize and tolerance, thereby reducing need for repeated propofol dose.

Better oxygenation outcomes was seen across several studies. Opioids can reduce respiratory drive and cause hypoventilation, particularly when combined with propofol. Ketamine has lesser direct respiratory depressant effect at procedural doses and may preserve airway reflex. However, ketamine does not eliminate this airway risk, and its advantages may be attenuated by deeper sedation targets, obesity, upper endoscopic instrumentation or concurrent propofol escalation (43, 44). This explains reason for some studies showing little difference between regimens and apparent respiratory benefit was not uniform across all included trials.

The lower incidence of oxygen desaturation with propofol–ketamine may be attributable to the relatively preserved respiratory function associated with ketamine compared with propofol (45). Propofol can cause dose-dependent respiratory depression, reduced ventilatory drive, upper-airway muscle relaxation, and airway obstruction (46, 47). In contrast, ketamine generally preserves spontaneous ventilation and upper-airway reflexes at procedural doses and has less direct respiratory depressant effect. Its sympathomimetic activity may also help maintain airway and cardiovascular function (45). Thus, the addition of ketamine may provide adequate analgesia and reduce the amount of propofol required, thereby decreasing cumulative respiratory depression and the risk of oxygen desaturation. However, this potential advantage may be reduced with deeper sedation, higher propofol doses, obesity, upper gastrointestinal instrumentation, or other factors affecting airway patency.

This review has several limitations. First, substantial clinical and methodological heterogeneity existed across studies, including differences in patient age, procedure type, ASA status, sedation depth, drug doses, administration protocols, and monitoring practices. Importantly, definitions and assessment methods for key outcomes also varied, particularly for oxygen desaturation, hypotension, haemodynamic changes, and recovery time, which limits direct comparison between studies. Second, several studies had some concerns or high risk of bias, and some were non-randomized, reducing the certainty of the findings. Third, the included populations and sample sizes were variable, with both paediatric and adult patients represented, which may limit generalizability. Finally, because of this heterogeneity, quantitative meta-analysis and precise estimates of comparative treatment effects were not performed. Therefore, the findings should be interpreted as overall patterns rather than definitive estimates of benefit or harm.

This review has important implications. Propofol-ketamine combination requires serious consideration as sedation strategy for gastrointestinal endoscopy, particularly in situations where maintenance of blood pressure, avoidance of oxygen desaturation or reduction of additional propofol dosing is necessary. This is especially relevant for longer procedures or more stimulating procedures, patients vulnerable to hypotension, and settings in which repeated propofol supplementation is not desirable. Better hemodynamic profile associated with propofol-ketamine may also be important for older adults, patients with limited cardiovascular reserve, and procedures in which blood pressure instability could compromise safety or workflow. However, clinicians need to keep in mind that recovery may be longer in certain patient groups or doses. This is important in high-throughput ambulatory units where rapid discharge is given major importance. Therefore, choice between propofol-ketamine and propofol-fentanyl should be guided by relative priority placed on intraprocedural stability against postprocedural recovery efficiency.

In clinical practice, these findings may help clinicians select the adjunct to propofol according to patient and procedural priorities. Propofol–ketamine may be considered when reducing propofol requirements and maintaining respiratory or hemodynamic stability are important, particularly during longer or more stimulating procedures. Propofol–fentanyl may remain an appropriate option when rapid recovery is prioritized and adequate respiratory monitoring is available. The choice should be individualized according to patient characteristics, procedure type, sedation requirements, and local expertise, with appropriate monitoring and readiness to manage airway, respiratory, or hemodynamic complications.

## Conclusion

This review suggests that propofol-ketamine combination provides better intraprocedural profile than propofol-fentanyl for gastrointestinal endoscopic sedation. This was particularly noticeable in outcomes like reduced propofol requirement, fewer desaturation events in many studies, and better preservation of systolic and mean arterial pressure. Overall, evidence indicates that ketamine might be valuable adjunct to combine with propofol when respiratory and hemodynamic stability are main priorities. However, recovery related trade offs and study heterogeneity indicates that regimen selection should remain individualized.

## Data Availability

The data analyzed in this study is subject to the following licenses/restrictions: the data will be shared on a reasonable request by author. Requests to access these datasets should be directed to email: saramedical@sina.com.
