# Supplementary material for: Comparison of propofol fentanyl versus propofol ketamine for painless gastrointestinal endoscopy: a systematic review
Source: Front Med (Lausanne). 2026 Sep 21;13:1946236. doi: 10.3389/fmed.2026.1946236 (PMC13634828; doi:10.3389/fmed.2026.1946236)
Supplement: Supplementary file 1 [file Supplementaryfile1.pdf]

Supplementary Appendix:

Search Strategy:

PubMed/MEDLINE: (("Endoscopy, Gastrointestinal"[Mesh] OR "Colonoscopy"[Mesh] OR "Gastroscopy"[Mesh] OR "Sigmoidoscopy"[Mesh] OR "Esophagoscopy"[Mesh] OR "Cholangiopancreatography, Endoscopic Retrograde"[Mesh] OR "Endosonography"[Mesh] OR "Gastrostomy"[Mesh] OR "gastrointestinal endoscopy"[tiab] OR "gastrointestinal endoscopic"[tiab] OR "gastrointestinal endoscopies"[tiab] OR "GI endoscopy"[tiab] OR "GI endoscopic"[tiab] OR "GIT endoscopy"[tiab] OR "digestive endoscopy"[tiab] OR "digestive endoscopic"[tiab] OR endoscopy[tiab] OR endoscopic[tiab] OR endoscopies[tiab] OR "upper gastrointestinal endoscopy"[tiab] OR "upper GI endoscopy"[tiab] OR "upper GIT endoscopy"[tiab] OR "upper endoscopy"[tiab] OR "upper digestive endoscopy"[tiab] OR "lower gastrointestinal endoscopy"[tiab] OR "lower GI endoscopy"[tiab] OR "lower GIT endoscopy"[tiab] OR "lower endoscopy"[tiab] OR gastroscopy[tiab] OR gastroscopic[tiab] OR gastroscopies[tiab] OR esophagogastroduodenoscopy[tiab] OR oesophagogastroduodenoscopy[tiab] OR esophago-gastro-duodenoscopy[tiab] OR oesophago-gastro-duodenoscopy[tiab] OR EGD[tiab] OR OGD[tiab] OR UGIE[tiab] OR UGI[tiab] OR colonoscopy[tiab] OR colonoscopic[tiab] OR colonoscopies[tiab] OR sigmoidoscopy[tiab] OR sigmoidoscopic[tiab] OR rectosigmoidoscopy[tiab] OR proctosigmoidoscopy[tiab] OR enteroscopy[tiab] OR enteroscopic[tiab] OR duodenoscopy[tiab] OR duodenoscopic[tiab] OR "double balloon enteroscopy"[tiab] OR "single balloon enteroscopy"[tiab] OR "balloon assisted enteroscopy"[tiab] OR "endoscopic retrograde cholangiopancreatography"[tiab] OR "endoscopic retrograde cholangio-pancreatography"[tiab] OR ERCP[tiab] OR cholangiopancreatography[tiab] OR "endoscopic ultrasound"[tiab] OR endosonography[tiab] OR EUS[tiab] OR "endoscopic ultrasonography"[tiab] OR "percutaneous endoscopic gastrostomy"[tiab] OR PEG[tiab] OR

"diagnostic endoscopy"[tiab] OR "therapeutic endoscopy"[tiab] OR "pediatric endoscopy"[tiab] OR "paediatric endoscopy"[tiab] OR "pediatric gastrointestinal endoscopy"[tiab] OR "paediatric gastrointestinal endoscopy"[tiab]) AND ("Sedation"[Mesh] OR "Conscious Sedation"[Mesh] OR "Deep Sedation"[Mesh] OR "Anesthesia"[Mesh] OR "Anesthesia, Intravenous"[Mesh] OR "Anesthetics, Intravenous"[Mesh] OR "Analgesia"[Mesh] OR sedation[tiab] OR sedative[tiab] OR sedatives[tiab] OR sedated[tiab] OR sedating[tiab] OR sedoanalgesia[tiab] OR sedo-analgesia[tiab] OR analgosedation[tiab] OR analgo-sedation[tiab] OR analgesia[tiab] OR analgesic[tiab] OR analgesics[tiab] OR anesthesia[tiab] OR anaesthesia[tiab] OR anesthetic[tiab] OR anaesthetic[tiab] OR anesthetics[tiab] OR anaesthetics[tiab] OR "procedural sedation"[tiab] OR "procedural sedation and analgesia"[tiab] OR PSA[tiab] OR "conscious sedation"[tiab] OR "moderate sedation"[tiab] OR "deep sedation"[tiab] OR "intravenous sedation"[tiab] OR "IV sedation"[tiab] OR "monitored anesthesia care"[tiab] OR "monitored anaesthesia care"[tiab] OR MAC[tiab] OR "balanced propofol sedation"[tiab] OR "endoscopic sedation"[tiab] OR "sedation during endoscopy"[tiab] OR "sedation for endoscopy"[tiab] OR "painless endoscopy"[tiab] OR "painless gastrointestinal endoscopy"[tiab] OR "non-anesthesiologist administered sedation"[tiab] OR "non-anaesthesiologist administered sedation"[tiab] OR "nonanesthesiologist administered sedation"[tiab] OR "nonanaesthesiologist administered sedation"[tiab] OR "non-anesthetist administered sedation"[tiab] OR "non-anaesthetist administered sedation"[tiab] OR NAPS[tiab] OR NAAP[tiab]) AND ("Propofol"[Mesh] OR propofol[tiab] OR diprivan[tiab] OR disoprofol[tiab] OR "2,6-diisopropylphenol"[tiab] OR "2 6 diisopropylphenol"[tiab] OR "propofol based"[tiab] OR "propofol-based"[tiab] OR "propofol sedation"[tiab] OR "propofol anesthesia"[tiab] OR "propofol anaesthesia"[tiab] OR ketofol[tiab] OR fenofol[tiab]) AND ("Ketamine"[Mesh] OR ketamine[tiab] OR ketamin[tiab] OR ketofol[tiab] OR "keta-fol"[tiab] OR "ketamine propofol"[tiab] OR

"ketamine-propofol"[tiab] OR "ketamine/propofol"[tiab] OR "ketamine plus propofol"[tiab] OR "ketamine and propofol"[tiab] OR "propofol ketamine"[tiab] OR "propofol-ketamine"[tiab] OR "propofol/ketamine"[tiab] OR "propofol plus ketamine"[tiab] OR "propofol and ketamine"[tiab] OR "PK combination"[tiab] OR esketamine[tiab] OR "S-ketamine"[tiab] OR "s ketamine"[tiab]) AND ("Fentanyl"[Mesh] OR fentanyl[tiab] OR fentanil[tiab] OR "fentanyl citrate"[tiab] OR "fentanil citrate"[tiab] OR sublimaze[tiab] OR fenofol[tiab] OR "fentanyl propofol"[tiab] OR "fentanyl-propofol"[tiab] OR "fentanyl/propofol"[tiab] OR "fentanyl plus propofol"[tiab] OR "fentanyl and propofol"[tiab] OR "propofol fentanyl"[tiab] OR "propofol-fentanyl"[tiab] OR "propofol/fentanyl"[tiab] OR "propofol plus fentanyl"[tiab] OR "propofol and fentanyl"[tiab] OR "PF combination"[tiab] OR "opioid propofol"[tiab] OR "opioid-propofol"[tiab] OR "opioid based sedation"[tiab] OR "opioid-based sedation"[tiab]))

Scopus: TITLE-ABS-KEY(("gastrointestinal endoscop*" OR "GI endoscop*" OR "GIT endoscop*" OR "digestive endoscop*" OR endoscop* OR "upper gastrointestinal endoscop*" OR "upper GI endoscop*" OR "upper GIT endoscop*" OR "upper endoscop*" OR "upper digestive endoscop*" OR "lower gastrointestinal endoscop*" OR "lower GI endoscop*" OR "lower GIT endoscop*" OR "lower endoscop*" OR gastroscop* OR esophagogastroduodenoscop* OR oesophagogastroduodenoscop* OR "esophago-gastro-duodenoscop*" OR "oesophago-gastro-duodenoscop*" OR EGD OR OGD OR UGIE OR UGI OR colonoscop* OR sigmoidoscop* OR rectosigmoidoscop* OR proctosigmoidoscop* OR enteroscop* OR duodenoscop* OR "double balloon enteroscop*" OR "single balloon enteroscop*" OR "balloon assisted enteroscop*" OR "endoscopic retrograde cholangiopancreatograph*" OR "endoscopic retrograde cholangio-pancreatograph*" OR ERCP OR cholangiopancreatograph* OR "endoscopic ultrasound" OR "endoscopic ultrasonograph*" OR endosonograph* OR EUS OR "percutaneous endoscopic gastrostom*"

OR PEG OR "diagnostic endoscop*" OR "therapeutic endoscop*" OR "pediatric endoscop*" OR "paediatric endoscop*" OR "pediatric gastrointestinal endoscop*" OR "paediatric gastrointestinal endoscop*") AND (sedat* OR sedative* OR sedoanalges* OR "sedo-analges*" OR analgosedat* OR "analgo-sedat*" OR analges* OR anesthe* OR anaesthe* OR "procedural sedation" OR "procedural sedation and analgesia" OR PSA OR "conscious sedation" OR "moderate sedation" OR "deep sedation" OR "intravenous sedation" OR "IV sedation" OR "monitored anesthesia care" OR "monitored anaesthesia care" OR MAC OR "balanced propofol sedation" OR "endoscopic sedation" OR "sedation during endoscop*" OR "sedation for endoscop*" OR "painless endoscop*" OR "painless gastrointestinal endoscop*" OR "non-anesthesiologist administered sedation" OR "non-anaesthesiologist administered sedation" OR "nonanesthesiologist administered sedation" OR "nonanaesthesiologist administered sedation" OR "non-anesthetist administered sedation" OR "non-anaesthetist administered sedation" OR NAPS OR NAAP) AND (propofol OR diprivan OR disoprofol OR "2,6-diisopropylphenol" OR "2 6 diisopropylphenol" OR "propofol based" OR "propofol-based" OR "propofol sedation" OR "propofol anesthesia" OR "propofol anaesthesia" OR ketofol OR fenofol) AND (ketamine OR ketamin OR ketofol OR "keta-fol" OR "ketamine propofol" OR "ketamine-propofol" OR "ketamine/propofol" OR "ketamine plus propofol" OR "ketamine and propofol" OR "propofol ketamine" OR "propofol-ketamine" OR "propofol/ketamine" OR "propofol plus ketamine" OR "propofol and ketamine" OR "PK combination" OR esketamine OR "S-ketamine" OR "s ketamine") AND (fentanyl OR fentanil OR "fentanyl citrate" OR "fentanil citrate" OR sublimaze OR fenofol OR "fentanyl propofol" OR "fentanyl-propofol" OR "fentanyl/propofol" OR "fentanyl plus propofol" OR "fentanyl and propofol" OR "propofol fentanyl" OR "propofol-fentanyl" OR "propofol/fentanyl" OR "propofol plus fentanyl" OR "propofol and fentanyl" OR "PF combination" OR "opioid propofol" OR "opioid-propofol" OR "opioid based sedation" OR "opioid-based sedation"))

Embase: ('gastrointestinal endoscopy'/exp OR 'colonoscopy'/exp OR 'gastroscopy'/exp OR 'sigmoidoscopy'/exp OR 'esophagoscopy'/exp OR 'endoscopic retrograde cholangiopancreatography'/exp OR 'endoscopic ultrasonography'/exp OR 'endosonography'/exp OR 'percutaneous endoscopic gastrostomy'/exp OR 'gastrointestinal endoscopy':ti,ab,kw OR 'gastrointestinal endoscopic':ti,ab,kw OR 'gastrointestinal endoscopies':ti,ab,kw OR 'gi endoscopy':ti,ab,kw OR 'gi endoscopic':ti,ab,kw OR 'git endoscopy':ti,ab,kw OR 'digestive endoscopy':ti,ab,kw OR 'digestive endoscopic':ti,ab,kw OR endoscopy:ti,ab,kw OR endoscopic:ti,ab,kw OR endoscopies:ti,ab,kw OR 'upper gastrointestinal endoscopy':ti,ab,kw OR 'upper gi endoscopy':ti,ab,kw OR 'upper git endoscopy':ti,ab,kw OR 'upper endoscopy':ti,ab,kw OR 'upper digestive endoscopy':ti,ab,kw OR 'lower gastrointestinal endoscopy':ti,ab,kw OR 'lower gi endoscopy':ti,ab,kw OR 'lower git endoscopy':ti,ab,kw OR 'lower endoscopy':ti,ab,kw OR gastroscopy:ti,ab,kw OR gastroscopic:ti,ab,kw OR gastroscopies:ti,ab,kw OR esophagogastroduodenoscopy:ti,ab,kw OR oesophagogastroduodenoscopy:ti,ab,kw OR 'esophago-gastro-duodenoscopy':ti,ab,kw OR 'oesophago-gastro-duodenoscopy':ti,ab,kw OR egd:ti,ab,kw OR ogd:ti,ab,kw OR ugie:ti,ab,kw OR ugi:ti,ab,kw OR colonoscopy:ti,ab,kw OR colonoscopic:ti,ab,kw OR colonoscopies:ti,ab,kw OR sigmoidoscopy:ti,ab,kw OR sigmoidoscopic:ti,ab,kw OR rectosigmoidoscopy:ti,ab,kw OR proctosigmoidoscopy:ti,ab,kw OR enteroscopy:ti,ab,kw OR enteroscopic:ti,ab,kw OR duodenoscopy:ti,ab,kw OR duodenoscopic:ti,ab,kw OR 'double balloon enteroscopy':ti,ab,kw OR 'single balloon enteroscopy':ti,ab,kw OR 'balloon assisted enteroscopy':ti,ab,kw OR 'endoscopic retrograde cholangiopancreatography':ti,ab,kw OR 'endoscopic retrograde cholangio-pancreatography':ti,ab,kw OR ercp:ti,ab,kw OR cholangiopancreatography:ti,ab,kw OR 'endoscopic ultrasound':ti,ab,kw OR 'endoscopic ultrasonography':ti,ab,kw OR endosonography:ti,ab,kw OR eus:ti,ab,kw OR 'percutaneous endoscopic gastrostomy':ti,ab,kw OR peg:ti,ab,kw OR 'diagnostic endoscopy':ti,ab,kw OR

'therapeutic endoscopy':ti,ab,kw OR 'pediatric endoscopy':ti,ab,kw OR 'paediatric endoscopy':ti,ab,kw OR 'pediatric gastrointestinal endoscopy':ti,ab,kw OR 'paediatric gastrointestinal endoscopy':ti,ab,kw) AND ('sedation'/exp OR 'conscious sedation'/exp OR 'deep sedation'/exp OR 'anesthesia'/exp OR 'intravenous anesthesia'/exp OR 'intravenous anesthetic agent'/exp OR 'analgesia'/exp OR sedation:ti,ab,kw OR sedative*:ti,ab,kw OR sedated:ti,ab,kw OR sedating:ti,ab,kw OR sedoanalgesia:ti,ab,kw OR 'sedo-analgesia':ti,ab,kw OR analgosedation:ti,ab,kw OR 'analgo-sedation':ti,ab,kw OR analgesia:ti,ab,kw OR analgesic*:ti,ab,kw OR anesthesia:ti,ab,kw OR anaesthesia:ti,ab,kw OR anesthetic*:ti,ab,kw OR anaesthetic*:ti,ab,kw OR 'procedural sedation':ti,ab,kw OR 'procedural sedation and analgesia':ti,ab,kw OR psa:ti,ab,kw OR 'conscious sedation':ti,ab,kw OR 'moderate sedation':ti,ab,kw OR 'deep sedation':ti,ab,kw OR 'intravenous sedation':ti,ab,kw OR 'iv sedation':ti,ab,kw OR 'monitored anesthesia care':ti,ab,kw OR 'monitored anaesthesia care':ti,ab,kw OR mac:ti,ab,kw OR 'balanced propofol sedation':ti,ab,kw OR 'endoscopic sedation':ti,ab,kw OR 'sedation during endoscopy':ti,ab,kw OR 'sedation for endoscopy':ti,ab,kw OR 'painless endoscopy':ti,ab,kw OR 'painless gastrointestinal endoscopy':ti,ab,kw OR 'non-anesthesiologist administered sedation':ti,ab,kw OR 'non-anaesthesiologist administered sedation':ti,ab,kw OR 'nonanesthesiologist administered sedation':ti,ab,kw OR 'nonanaesthesiologist administered sedation':ti,ab,kw OR 'non-anesthetist administered sedation':ti,ab,kw OR 'non-anaesthetist administered sedation':ti,ab,kw OR naps:ti,ab,kw OR naap:ti,ab,kw) AND ('propofol'/exp OR propofol:ti,ab,kw OR diprivan:ti,ab,kw OR disoprofol:ti,ab,kw OR '2,6-diisopropylphenol':ti,ab,kw OR '2 6 diisopropylphenol':ti,ab,kw OR 'propofol based':ti,ab,kw OR 'propofol-based':ti,ab,kw OR 'propofol sedation':ti,ab,kw OR 'propofol anesthesia':ti,ab,kw OR 'propofol anaesthesia':ti,ab,kw OR ketofol:ti,ab,kw OR fenofol:ti,ab,kw) AND ('ketamine'/exp OR 'esketamine'/exp OR ketamine:ti,ab,kw OR

ketamin:ti,ab,kw OR ketofol:ti,ab,kw OR 'keta-fol':ti,ab,kw OR 'ketamine propofol':ti,ab,kw OR 'ketamine-propofol':ti,ab,kw OR 'ketamine/propofol':ti,ab,kw OR 'ketamine plus propofol':ti,ab,kw OR 'ketamine and propofol':ti,ab,kw OR 'propofol ketamine':ti,ab,kw OR 'propofol-ketamine':ti,ab,kw OR 'propofol/ketamine':ti,ab,kw OR 'propofol plus ketamine':ti,ab,kw OR 'propofol and ketamine':ti,ab,kw OR 'pk combination':ti,ab,kw OR esketamine:ti,ab,kw OR 's-ketamine':ti,ab,kw OR 's ketamine':ti,ab,kw) AND ('fentanyl'/exp OR fentanyl:ti,ab,kw OR fentanil:ti,ab,kw OR 'fentanyl citrate':ti,ab,kw OR 'fentanil citrate':ti,ab,kw OR sublimaze:ti,ab,kw OR fenofol:ti,ab,kw OR 'fentanyl propofol':ti,ab,kw OR 'fentanyl-propofol':ti,ab,kw OR 'fentanyl/propofol':ti,ab,kw OR 'fentanyl plus propofol':ti,ab,kw OR 'fentanyl and propofol':ti,ab,kw OR 'propofol fentanyl':ti,ab,kw OR 'propofol-fentanyl':ti,ab,kw OR 'propofol/fentanyl':ti,ab,kw OR 'propofol plus fentanyl':ti,ab,kw OR 'propofol and fentanyl':ti,ab,kw OR 'pf combination':ti,ab,kw OR 'opioid propofol':ti,ab,kw OR 'opioid-propofol':ti,ab,kw OR 'opioid based sedation':ti,ab,kw OR 'opioid-based sedation':ti,ab,kw)

Cochrane CENTRAL: ([mh "Endoscopy, Gastrointestinal"] OR [mh Colonoscopy] OR [mh Gastroscopy] OR [mh Sigmoidoscopy] OR [mh Esophagoscopy] OR [mh "Cholangiopancreatography, Endoscopic Retrograde"] OR [mh Endosonography] OR [mh Gastrostomy] OR "gastrointestinal endoscopy":ti,ab,kw OR "gastrointestinal endoscopic":ti,ab,kw OR "gastrointestinal endoscopies":ti,ab,kw OR "GI endoscopy":ti,ab,kw OR "GI endoscopic":ti,ab,kw OR "GIT endoscopy":ti,ab,kw OR "digestive endoscopy":ti,ab,kw OR "digestive endoscopic":ti,ab,kw OR endoscopy:ti,ab,kw OR endoscopic:ti,ab,kw OR endoscopies:ti,ab,kw OR "upper gastrointestinal endoscopy":ti,ab,kw OR "upper GI endoscopy":ti,ab,kw OR "upper GIT endoscopy":ti,ab,kw OR "upper endoscopy":ti,ab,kw OR "upper digestive endoscopy":ti,ab,kw OR "lower gastrointestinal endoscopy":ti,ab,kw OR "lower GI endoscopy":ti,ab,kw OR "lower GIT

endoscopy":ti,ab,kw OR "lower endoscopy":ti,ab,kw OR gastroscopy:ti,ab,kw OR gastroscopic:ti,ab,kw OR gastroscopies:ti,ab,kw OR esophagogastroduodenoscopy:ti,ab,kw OR oesophagogastroduodenoscopy:ti,ab,kw OR "esophago-gastro-duodenoscopy":ti,ab,kw OR "oesophago-gastro-duodenoscopy":ti,ab,kw OR EGD:ti,ab,kw OR OGD:ti,ab,kw OR UGIE:ti,ab,kw OR UGI:ti,ab,kw OR colonoscopy:ti,ab,kw OR colonoscopic:ti,ab,kw OR colonoscopies:ti,ab,kw OR sigmoidoscopy:ti,ab,kw OR sigmoidoscopic:ti,ab,kw OR rectosigmoidoscopy:ti,ab,kw OR proctosigmoidoscopy:ti,ab,kw OR enteroscopy:ti,ab,kw OR enteroscopic:ti,ab,kw OR duodenoscopy:ti,ab,kw OR duodenoscopic:ti,ab,kw OR "double balloon enteroscopy":ti,ab,kw OR "single balloon enteroscopy":ti,ab,kw OR "balloon assisted enteroscopy":ti,ab,kw OR "endoscopic retrograde cholangiopancreatography":ti,ab,kw OR "endoscopic retrograde cholangio-pancreatography":ti,ab,kw OR ERCP:ti,ab,kw OR cholangiopancreatography:ti,ab,kw OR "endoscopic ultrasound":ti,ab,kw OR "endoscopic ultrasonography":ti,ab,kw OR endosonography:ti,ab,kw OR EUS:ti,ab,kw OR "percutaneous endoscopic gastrostomy":ti,ab,kw OR PEG:ti,ab,kw OR "diagnostic endoscopy":ti,ab,kw OR "therapeutic endoscopy":ti,ab,kw OR "pediatric endoscopy":ti,ab,kw OR "paediatric endoscopy":ti,ab,kw OR "pediatric gastrointestinal endoscopy":ti,ab,kw OR "paediatric gastrointestinal endoscopy":ti,ab,kw) AND ([mh Sedation] OR [mh "Conscious Sedation"] OR [mh "Deep Sedation"] OR [mh Anesthesia] OR [mh "Anesthesia, Intravenous"] OR [mh "Anesthetics, Intravenous"] OR [mh Analgesia] OR sedation:ti,ab,kw OR sedative*:ti,ab,kw OR sedated:ti,ab,kw OR sedating:ti,ab,kw OR sedoanalgesia:ti,ab,kw OR "sedo-analgesia":ti,ab,kw OR analgosedation:ti,ab,kw OR "analgo-sedation":ti,ab,kw OR analgesia:ti,ab,kw OR analgesic*:ti,ab,kw OR anesthesia:ti,ab,kw OR anaesthesia:ti,ab,kw OR anesthetic*:ti,ab,kw OR anaesthetic*:ti,ab,kw OR "procedural sedation":ti,ab,kw OR "procedural sedation and analgesia":ti,ab,kw OR PSA:ti,ab,kw OR "conscious sedation":ti,ab,kw OR "moderate sedation":ti,ab,kw OR "deep sedation":ti,ab,kw OR

"intravenous sedation":ti,ab,kw OR "IV sedation":ti,ab,kw OR "monitored anesthesia care":ti,ab,kw OR "monitored anaesthesia care":ti,ab,kw OR MAC:ti,ab,kw OR "balanced propofol sedation":ti,ab,kw OR "endoscopic sedation":ti,ab,kw OR "sedation during endoscopy":ti,ab,kw OR "sedation for endoscopy":ti,ab,kw OR "painless endoscopy":ti,ab,kw OR "painless gastrointestinal endoscopy":ti,ab,kw OR "non-anesthesiologist administered sedation":ti,ab,kw OR "non-anaesthesiologist administered sedation":ti,ab,kw OR "nonanesthesiologist administered sedation":ti,ab,kw OR "nonanaesthesiologist administered sedation":ti,ab,kw OR "non-anesthetist administered sedation":ti,ab,kw OR "non-anaesthetist administered sedation":ti,ab,kw OR NAPS:ti,ab,kw OR NAAP:ti,ab,kw) AND ([mh Propofol] OR propofol:ti,ab,kw OR diprivan:ti,ab,kw OR disoprofol:ti,ab,kw OR "2,6-diisopropylphenol":ti,ab,kw OR "2 6 diisopropylphenol":ti,ab,kw OR "propofol based":ti,ab,kw OR "propofol-based":ti,ab,kw OR "propofol sedation":ti,ab,kw OR "propofol anesthesia":ti,ab,kw OR "propofol anaesthesia":ti,ab,kw OR ketofol:ti,ab,kw OR fenofol:ti,ab,kw) AND ([mh Ketamine] OR ketamine:ti,ab,kw OR ketamin:ti,ab,kw OR ketofol:ti,ab,kw OR "keta-fol":ti,ab,kw OR "ketamine propofol":ti,ab,kw OR "ketamine-propofol":ti,ab,kw OR "ketamine/propofol":ti,ab,kw OR "ketamine plus propofol":ti,ab,kw OR "ketamine and propofol":ti,ab,kw OR "propofol ketamine":ti,ab,kw OR "propofol-ketamine":ti,ab,kw OR "propofol/ketamine":ti,ab,kw OR "propofol plus ketamine":ti,ab,kw OR "propofol and ketamine":ti,ab,kw OR "PK combination":ti,ab,kw OR esketamine:ti,ab,kw OR "S-ketamine":ti,ab,kw OR "s ketamine":ti,ab,kw) AND ([mh Fentanyl] OR fentanyl:ti,ab,kw OR fentanil:ti,ab,kw OR "fentanyl citrate":ti,ab,kw OR "fentanil citrate":ti,ab,kw OR sublimaze:ti,ab,kw OR fenofol:ti,ab,kw OR "fentanyl propofol":ti,ab,kw OR "fentanyl-propofol":ti,ab,kw OR "fentanyl/propofol":ti,ab,kw OR "fentanyl plus propofol":ti,ab,kw OR "fentanyl and propofol":ti,ab,kw OR "propofol fentanyl":ti,ab,kw OR "propofol-

fentanyl":ti,ab,kw OR "propofol/fentanyl":ti,ab,kw OR "propofol plus fentanyl":ti,ab,kw OR "propofol and fentanyl":ti,ab,kw OR "PF combination":ti,ab,kw OR "opioid propofol":ti,ab,kw OR "opioid-propofol":ti,ab,kw OR "opioid based sedation":ti,ab,kw OR "opioid-based sedation":ti,ab,kw)

Web of Science: TS=(("gastrointestinal endoscop*" OR "GI endoscop*" OR "GIT endoscop*" OR "digestive endoscop*" OR endoscop* OR "upper gastrointestinal endoscop*" OR "upper GI endoscop*" OR "upper GIT endoscop*" OR "upper endoscop*" OR "upper digestive endoscop*" OR "lower gastrointestinal endoscop*" OR "lower GI endoscop*" OR "lower GIT endoscop*" OR "lower endoscop*" OR gastroscop* OR esophagogastroduodenoscop* OR oesophagogastroduodenoscop* OR "esophago-gastro-duodenoscop*" OR "oesophago-gastro-duodenoscop*" OR EGD OR OGD OR UGIE OR UGI OR colonoscop* OR sigmoidoscop* OR rectosigmoidoscop* OR proctosigmoidoscop* OR enteroscop* OR duodenoscop* OR "double balloon enteroscop*" OR "single balloon enteroscop*" OR "balloon assisted enteroscop*" OR "endoscopic retrograde cholangiopancreatograph*" OR "endoscopic retrograde cholangio-pancreatograph*" OR ERCP OR cholangiopancreatograph* OR "endoscopic ultrasound" OR "endoscopic ultrasonograph*" OR endosonograph* OR EUS OR "percutaneous endoscopic gastrostom*" OR PEG OR "diagnostic endoscop*" OR "therapeutic endoscop*" OR "pediatric endoscop*" OR "paediatric endoscop*" OR "pediatric gastrointestinal endoscop*" OR "paediatric gastrointestinal endoscop*") AND (sedat* OR sedative* OR sedoanalges* OR "sedo-analges*" OR analgosedat* OR "analgo-sedat*" OR analges* OR anesthe* OR anaesthe* OR "procedural sedation" OR "procedural sedation and analgesia" OR PSA OR "conscious sedation" OR "moderate sedation" OR "deep sedation" OR "intravenous sedation" OR "IV sedation" OR "monitored anesthesia care" OR "monitored anaesthesia care" OR MAC OR "balanced propofol sedation" OR "endoscopic sedation" OR "sedation during endoscop*" OR

"sedation for endoscop*" OR "painless endoscop*" OR "painless gastrointestinal endoscop*" OR "non-anesthesiologist administered sedation" OR "non-anaesthesiologist administered sedation" OR "nonanesthesiologist administered sedation" OR "nonanaesthesiologist administered sedation" OR "non-anesthetist administered sedation" OR "non-anaesthetist administered sedation" OR NAPS OR NAAP) AND (propofol OR diprivan OR disoprofol OR "2,6-diisopropylphenol" OR "2 6 diisopropylphenol" OR "propofol based" OR "propofol-based" OR "propofol sedation" OR "propofol anesthesia" OR "propofol anaesthesia" OR ketofol OR fenofol) AND (ketamine OR ketamin OR ketofol OR "keta-fol" OR "ketamine propofol" OR "ketamine-propofol" OR "ketamine/propofol" OR "ketamine plus propofol" OR "ketamine and propofol" OR "propofol ketamine" OR "propofol-ketamine" OR "propofol/ketamine" OR "propofol plus ketamine" OR "propofol and ketamine" OR "PK combination" OR esketamine OR "S-ketamine" OR "s ketamine") AND (fentanyl OR fentanil OR "fentanyl citrate" OR "fentanil citrate" OR sublimaze OR fenofol OR "fentanyl propofol" OR "fentanyl-propofol" OR "fentanyl/propofol" OR "fentanyl plus propofol" OR "fentanyl and propofol" OR "propofol fentanyl" OR "propofol-fentanyl" OR "propofol/fentanyl" OR "propofol plus fentanyl" OR "propofol and fentanyl" OR "PF combination" OR "opioid propofol" OR "opioid-propofol" OR "opioid based sedation" OR "opioid-based sedation"))
